# Active regression model for clinical grading of COVID-19

**DOI:** 10.3389/fimmu.2023.1141996

**Published:** 2023-03-21

**Authors:** Yuan Sh, Jierong Dong, Zhongqing Chen, Meiqing Yuan, Lingna Lyu, Xiuli Zhang

**Affiliations:** ^1^ Fujian Provincial Key Laboratory of Brain Aging and Neurodegenerative Diseases, School of Basic Medical Sciences, Fujian Medical University, Fuzhou, Fujian, China; ^2^ The Chinese Academy of Sciences (CAS) Key Laboratory for Biomedical Effects of Nanomaterials and Nanosafety, The Chinese Academy of Sciences (CAS) Key Laboratory of Standardization and Measurement for Nanotechnology, The Chinese Academy of Sciences (CAS) Center for Excellence in Nanoscience, National Center for Nanoscience and Technology of China, Beijing, China; ^3^ The First Affiliated Hospital of Fujian Medical University, Fuzhou, Fujian, China; ^4^ Key Laboratory of Forensic Genetics, Institute of Forensic Sciences, Ministry of Public Security, Beijing, China; ^5^ Department of Gastroenterology and Hepatology, Beijing You’an Hospital, Capital Medical University, Beijing, China

**Keywords:** COVID-19, deep learning, active regression, feature engineering, clinical data

## Abstract

**Background:**

In the therapeutic process of COVID-19, the majority of indicators that physicians have for assisting treatment have come from clinical tests represented by proteins, metabolites, and immune levels in patients’ blood. Therefore, this study constructs an individualized treatment model based on deep learning methods, aiming to realize timely intervention based on clinical test indicator data of COVID-19 patients and provide an important theoretical basis for optimizing medical resource allocation.

**Methods:**

This study collected clinical data from a total of 1,799 individuals, including 560 controls for non-respiratory infectious diseases (Negative), 681 controls for other respiratory virus infections (Other), and 558 coronavirus infections (Positive) for COVID-19. We first used the Student T-test to screen for statistically significant differences (Pvalue<0.05); we then used the Adaptive-Lasso method stepwise regression to screen the characteristic variables and filter the features with low importance; we then used analysis of covariance to calculate the correlation between variables and filter the highly correlated features; and finally, we analyzed the feature contribution and screened the best combination of features.

**Results:**

Feature engineering reduced the feature set to 13 feature combinations. The correlation coefficient between the projected results of the artificial intelligence-based individualized diagnostic model and the fitted curve of the actual values in the test group was 0.9449 which could be applied to the clinical prognosis of COVID-19. In addition, the depletion of platelets in patients with COVID-19 is an important factor affecting their severe deterioration. With the progression of COVID-19, there is a slight decrease in the total number of platelets in the patient’s body, particularly as the volume of larger platelets sharply decreases. The importance of plateletCV (count*mean platelet volume) in evaluating the severity of COVID-19 patients is higher than the count of platelets and mean platelet volume.

**Conclusion:**

In general, we found that for patients with COVID-19, the increase in mean platelet volume was a predictor for SARS-Cov-2. The rapid decrease of platelet volume and the decrease of total platelet volume are dangerous signals for the aggravation of SARS-Cov-2 infection. The analysis and modeling results of this study provide a new perspective for individualized accurate diagnosis and treatment of clinical COVID-19 patients.

## Introduction

Coronavirus disease 2019 (COVID-19) is an extremely contagious disease produced in humans by the severe acute respiratory syndrome coronavirus-2 (SARS-CoV-2) (1). Since the first instance of COVID-19 was found in December 2019, it has quickly spread throughout the entire planet (2–4). The World Health Organization classified COVID-19 as a “Global Pandemic” in March of 2020 (5). 81 percent of COVID-19 patients had cold-like symptoms and moderate pneumonia, 14 percent had severe respiratory syndrome, and 5 percent had critical respiratory failure, septic shock, and/or multiple organ dysfunction or failure; the overall fatality rate was 1%. In terms of severity and duration, COVID-19 has unique characteristics (6). In addition, the incidence of severe and critical cases is considerably greater than that of influenza and tuberculosis (7). Individual differences in gene expression, metabolite levels, and immune cell composition affect the immune response *via* numerous mechanisms and impact COVID-19 susceptibility, disease severity, and prognosis (8–10). Therefore, it is essential to investigate the pathophysiological characteristics of patients, identify risk factors of disease progression for clinical diagnosis, and create COVID-19 preventive and therapeutic measures (11, 12).

With the continuous optimization and improvement of computer technology, statistics, deep learning and other techniques are widely used for accurate data analysis (13–16). Deep learning-based artificial intelligence algorithms have recently demonstrated distinct advantages in the accurate detection and treatment of diseases (17–21). Using a multi-classification modeling approach with unique risk indices, this method simultaneously predicts numerous disease states and generates a model with a high level of accuracy (22, 23). Using CT images of COVID-19 patients, researchers from the Macau University of Science and Technology presented their findings on deep learning modeling in the journal Cell in June of 2020 (24). Overall, the system differentiated COVID-19 pneumonia patients from the other two groups (other common pneumonia and healthy controls) with a sensitivity of 94.93%, a specificity of 91.13%, and an AUC of 0.89. Active learning is a machine learning method that actively selects the most valuable samples for labeling. The goal is to achieve the best possible performance of the model using the smallest possible number of high-quality samples with annotation (25). Zongwei Zhou et al. significantly reduce the cost of annotating medical images by integrating active learning and migration learning. Compared to state-of-the-art random selection methods, the annotation cost can be reduced by at least half in three medical applications and by over 33% in natural image datasets (26).

However, the majority of convenient features available to clinicians in real-world situations are derived from clinical test findings, such as blood proteins (27), metabolites, immune cell counts, etc (28). This project enrolled patients with varying severity levels, collected individualized clinical testing results from them, and used deep learning multi-model combination technology based on neural networks to obtain an artificial intelligence individualized diagnosis and treatment model for COVID-19 with fewer features, high accuracy, and promoted clinical application by optimizing modeling parameters to achieve convenient and rapid monitoring of COVID-19 (29, 30).

## Materials and methods

### Data collection and data cleaning

In this research, we collected 1,799 samples, including 560 individuals in the control group of non-respiratory infectious diseases (Negative), 681 patients in the control group of other respiratory viral infections (Other) (table of patients’ viral infections, **Supplement Table 1**), and 558 patients in the group of coronavirus. To be more in line with the real world and the clinical application, we did not consider comorbidity, sex, and other chronic pulmonary disease from the patient subgroups.

The clinical features are included as follows: 1) Clinical hematology indicators: leukocytes, red blood cells, bone marrow indicators, coagulation factors, platelet functions, blood type, blood rheology indicators, etc. 2) Clinical body fluid indicators: body fluid routine examination, urine chemistry, urine cell types, urine crystalline types, etc. 3) Clinical chemistry indicators: various proteins, sugar and metabolite indicators, lipid and lipoprotein levels, cardiac, hepatobiliary and renal disease indicators, other enzyme indicators, inorganic substance determination and blood gas analysis, vitamins, amino acids, thyroid function indicators, various trace elements, free radicals, sex hormones, other hormones, etc. 4) Clinical immunology indicators: tumor-related antigens, lymphocyte and subpopulation classification, cytokine classes, allergen test results, etc. 5) Other indicators: such as clinical bacteriological test indicators, virology, antibiotic classes, gene mutations, drug resistance genes, etc.

### Feature engineering

In this paper, all statistical learning methods were performed based on R version 4.2.1. Machine learning and deep learning modeling was performed based on python version 3.9.12 and scikit-learn version 1.0.2. The main statistical learning methods include ANOVA (data conform to normal distribution), Wilcox rank sum test (data are discrete variables), and Fisher’s exact test (data are frequency variables). False discovery rate (FDR) correction was performed for all P values using the Benjamini-Hochberg method, and variables were considered to be statistically significantly different when FDR < 0.05. Variables with high significance and statistically significant differences were screened using the adaptive-Lasso regression method. Variables with covariance higher than 0.95 were filtered out using Pearson or Spearman correlation. To explain the impact and contribution of each clinical characteristic on the classification of SARS-CoV-2 positive patients, we screened variables using the Shapley additive explanation method (SHAP).

We serialized patients with different severity levels by numerical simulation. The aim is to use the information of patients with different severity levels as an important reference indicator. Specifically, the transformation of the three subgroups into three continuous variables is based on an intrinsic linear pattern between the three severity levels, with the numbers increasing in severity and increasing in magnitude. The three subgroups belong to the three classification questions. However, the patients in the three subgroups are progressive in severity. The event y that we want to predict is not simply a three-classification problem. Therefore, we use a set of numbers that model the linear relationship between the three y’s internally. and then use the optimal set of features x for artificial neural network-based regression modeling. The hidden association information between the event y to be predicted and between y and the feature set x can be explored and used to the maximum extent to obtain the optimal model. After careful and sufficient debugging, we obtain the optimal numerical simulation of the event to be predicted y. Thus, the three groupings are transformed into three continuous variables, and the numbers become larger as the severity increases step by step. The basis of our grouping is based on the official hospitalization indicators given. Group 1: no hospitalization, patient whose nucleic acid test result is positive and has some clinical symptoms but not be hospitalized. Group 2: regular hospitalization, patient who has some clinical symptoms related to COVID-19 and need hospitalization. Group 3: semi-intensive unit or intensive-care unit, in this group patient need more carefully management which is staffed and equipped to provide a higher level of care than a general ward or patient who has severe and life-threatening medical conditions.

### Build the neural network

After screening out the important features by the above steps, the obtained features with significant contribution to different category groups were analyzed by deep learning methods to build disease prediction models. It was optimal to construct deep neural network models for disease prognosis assessment using the optimal set of features. Firstly, the data set was divided into training, validation and test sets (8:1:1). Second, since the initial values of each feature span a large range, this difference will lead to an imbalance of weights among the features introduced during model construction, which will affect the performance of the model. Therefore, we zero-averaged the features of the training, validation, and test sets so that each feature has the same variance. We use SELU as the main activation function of the model in model training to prevent the gradient disappearance/explosion phenomenon; epoch is set to 100, and to prevent overfitting, we set Dropout to 0.5 and early stop, where the patient parameter is 30.

## Result

### Comparison of the clinical features between COVID-19 and other groups

**Figure 1:**
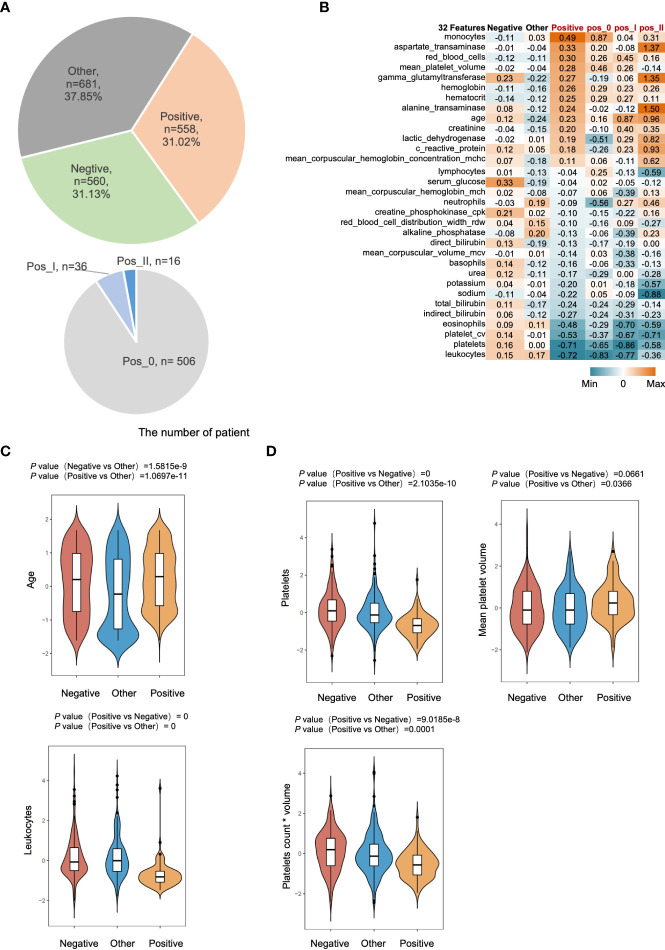
Distinct features of COVID-19. **(A)** The sample distribution in this study. Positive, represents confirmed COVID-19 infected patients. Negative, represents non-respiratory infectious patients. Other, represents non-COVID-19 infected patients (Top). Pos_0 represents latently infected patients. Pos_1, represents general inpatients. Pos_2, represents serious patients in the intensive care unit (Bottom). **(B)** The heatmap shows the relative expression level of 32 indicators with a cumulative contribution of 100% among different classification groups the number in heatmap is the average value within the group after zero mean normalization of the original expression value. **(C)** Box plot of normalize value of age and leukocytes among different classification groups. **(D)** Box plot of normalize value of platelets, mean platelet volume and platelets count * volume among different classification groups.

We collected a total of 1,799 individuals, including 560 controls for non-respiratory infectious diseases (Negative), 681 controls for other respiratory virus infections (Other), and 558 coronavirus infections (Positive) for COVID-19 (**Figure 1A**, Top). Clinical features, such as hematological indicators, chemical indicators, immunological indicators, and virological indicators, were gathered from each of the three groups to be compared with one another (**Supplementary Figure 1**). Negative, other, and COVID-19 were differentiated using a total of 32 distinct features (**Figure 1B**). When compared to other viruses that can cause respiratory viral infections (p = 1.0697e-11, **Figure 1C**), SARS-CoV-2 appears to preferentially infect older persons.

Alterations in patients’ coagulation levels have been found to have a strong correlation with a bad prognosis in COVID-19 patients (31–36). As a result, we analyzed platelet levels and their volumes in three groups and discovered that SARS-CoV-2 infection was linked with a decrease in platelet count but an increase in mean platelet volume. By calculating the plateletCV (count*mean platelet volume), we determined that COVID-19 had a significantly lower total platelet volume than the other two groups (**Figure 1D**).

### Development of three-phase COVID-19 individualized prediction model

**Figure 2:**
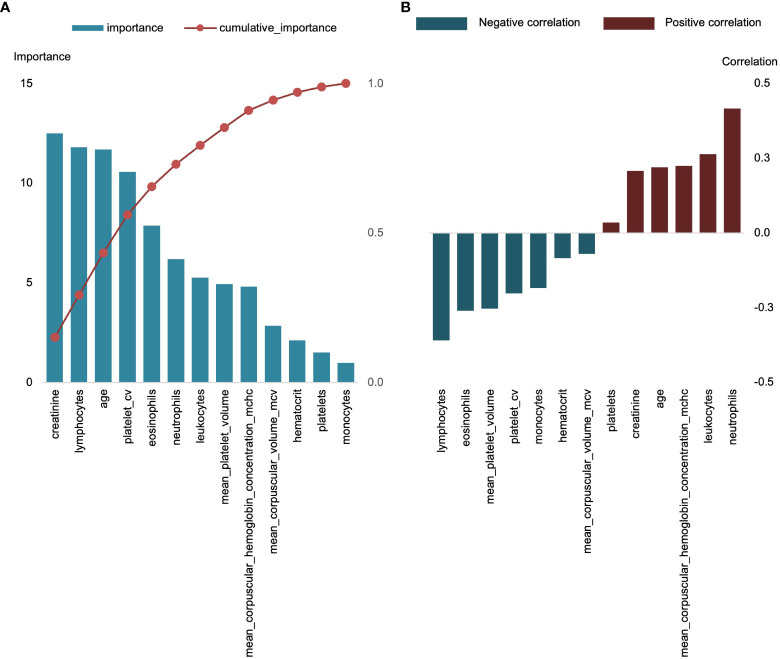
Top 13 features for classification of three-phase COVID-19. **(A)** The importance distribution and the cumulative importance curve of 13 features, left Y-axis represents importance, right Y-axis represents cumulative importance; X-axis is the name of each feature. **(B)** Correlation of 13 characteristics with positive group.

To examine the clinical characteristic indicators of diseases with different severity categories, we re-grouped the enrolled patients as control groups (mentioned above) and positive groups, and then classified them into three-phase diseases according to their severity as phase 0 (non-inpatients), phase I (general inpatients), and phase II (ICU inpatients), respectively. A total of 99 testing indexes of clinical features were uploaded as input to screen the variables by modeling importance analysis. The results showed that 13 features had a cumulative contribution of 100% in predicting the three-phase COVID-19 (**Figure 2A**), among which leukocytes levels, age, neutrophils levels, total platelet volume, mean hemoglobin concentration, and lymphocytes levels were most important. Interestingly, as disease progressed, features such as leukocytes levels, age, neutrophils levels, and mean hemoglobin concentration were obviously elevated (positive correlation), while total platelet volume, lymphocytes levels, and hemoglobin concentration were continuously reduced (negatively correlated) (**Figure 2B**).

**Figure 3:**
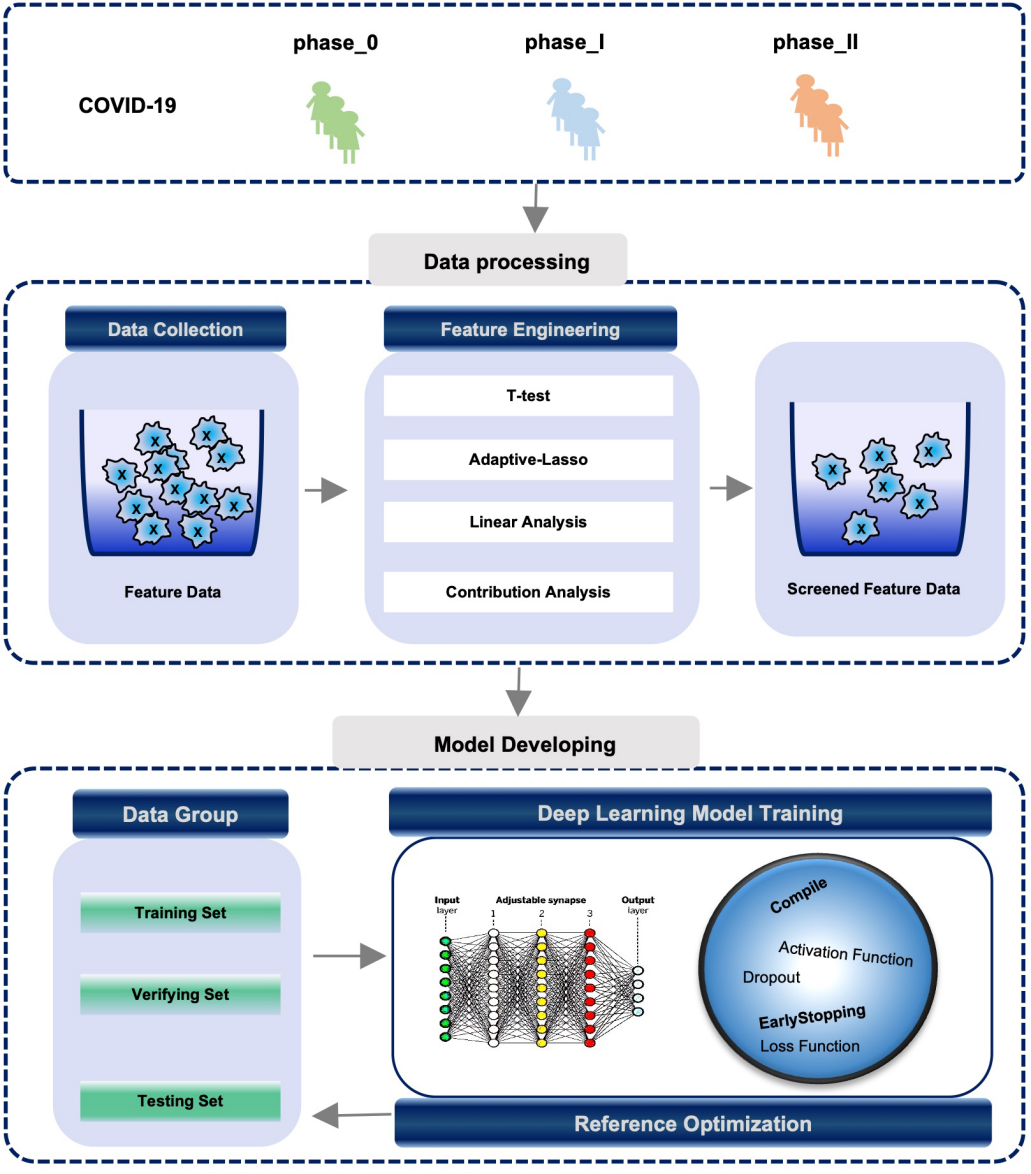
Process of constructing COVID-19 individualized diagnostic model based on active regression learning. Process of constructing a COVID-19 individualized diagnostic model based on active regression learning. (TOP) The samples are grouped into phase_0, phase_I, and phase_II according to different symptoms or disease types. (Middle) The optimal set of features is filtered using feature engineering methods (see Methods). (Bottom) Individualized diagnostic model building logic and model efficacy assessment rules.

**Figure 4:**
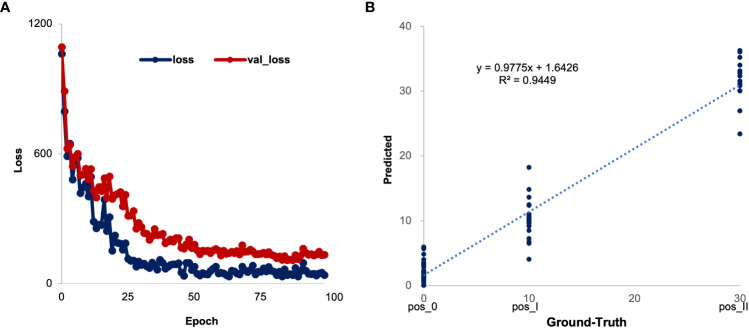
Process of constructing COVID-19 individualized diagnostic model based on active regression learning. **(A)** The change curve of the loss value of the training and validation sets during epoch iterations. The value of the loss function of the model is calculated from the mean squared error (MSE). **(B)** The test set results are used to evaluate the performance of model.

We employed 13 clinical features to construct a deep neural network model to explore the individualized prediction model for three distinct phases of COVID-19 (**Figure 3**). Using an iterative optimization method, the optimal model was chosen. The loss value for the training set is 31.08 and the validation set is 106.86. (**Figure 4A**). The severity of COVID-19 was then predicted using a linear model based on deep learning. The correlation coefficient between the projected results of the model and the fitting curve of the actual value is 0.9449 (**Figure 4B**), which may be applied to the COVID-19 clinical prognosis.

### Risk factors of severe COVID-19

**Figure 5:**
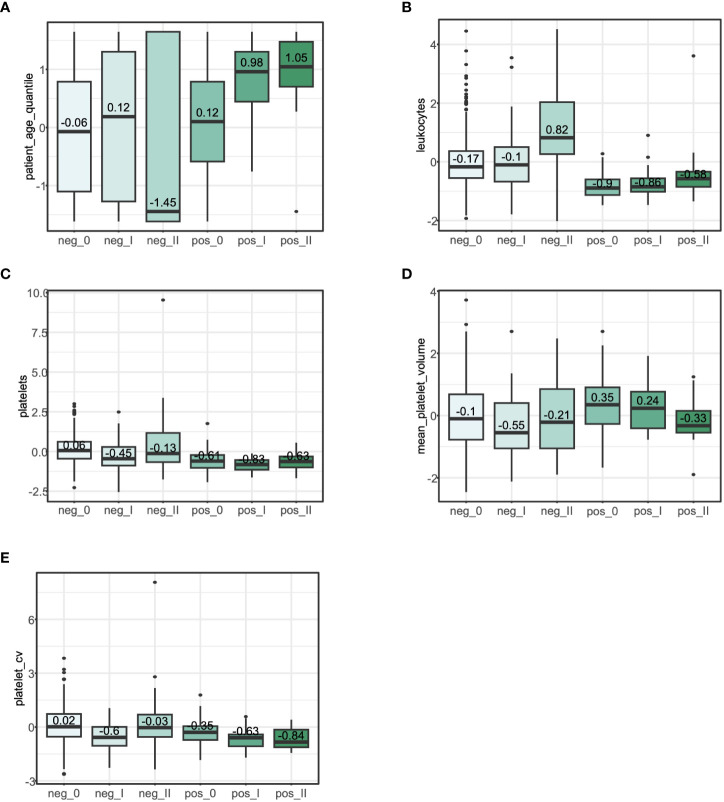
Risk factor analysis for three groups of COVID-19. pahse_0, latently infected; pahse_I, general inpatients; pahse_II, serious patients in ICU. Box plots show the differences in characteristics between different severity levels within different infection groups. Numbers are within-group means after zero-mean normalization of the original expression values. **(A)**, Box plot of the distribution of the age among different groups. **(B)**, Box plot of the distribution of the leukocyte among different groups. **(C)**, Box plot of the distribution of the number of total platelets among different groups. **(D)**, Box plot of the distribution of the mean platelet volume of platelets among different groups. **(E)**, Box plot of the distribution of the count*mean platelet volume among different groups.

To determine the correlation between coagulation levels and the severity of COVID-19, we classified SARS-CoV-2 positive patients into three phases: phase 0 patients were latently infected; phase I patients were general inpatients; and phase II patients were serious patients in the intensive care unit (ICU). As COVID-19 deteriorated, levels of total platelets in the patient’s blood declined somewhat, while the number of large platelets decreased substantially and the volume of total platelets in the blood continued to decrease (**Figure 5**). Differential analysis indicated that the overall age of the positive group was significantly higher compared to the control group. Severe COVID-19 (phase_II) were predominantly of advanced age, while other diseases (control group) generally lead to severe states in younger patients (**Figure 5A**). Leukocyte levels were slightly elevated in the severe COVID-19 (phase_II) compared to other less sever phases, but overall levels of leukocytes were lower than in the control group. (**Figure 5B**). However, leukocytes remained at a low level in patients after SARS-Cov-2 infection.

By comparing the levels of platelet-related features, we found that the level of platelets with large volume was dramatically reduced and the total platelet volume in blood was continuously decreased (**Figures 5C–E**) within progressing three-phase COVID-19, which was in accordance with our three-phase categories of COVID-19 in **Figure 1**. It has been reported that myocardial infarction patients with elevated mean platelet volumes had a significantly increased incidence of death and heart failure (37, 38).

## Discussion

Since the outbreak of SARS-Cov-2 virus in 2019, it has caused a huge impact on global health security and economic security. There has been an ongoing effort to develop artificial intelligence models for automated diagnosis and real-time surveillance of COVID-19 (39–43). In this study, we developed a simple, efficient, and clinically accessible AI model based on a large cohort population. The model uses fewer and easily accessible clinical features (N=13) to achieve high accuracy in patient prognosis assessment and monitoring (independent test set R^2 =^ 0.9449). Feature filtering and extraction of large-scale clinical multi-omics data using a feature engineering approach provided us with a clearer understanding of changes in clinical indicators in patients infected with SARS-CoV-2, such as significantly lower serum leukocyte counts in COVID-19 patients than in other groups; impaired coagulation-related function was an important risk factor for COVID-19 patients, especially the mean platelet volume of rapid reduction. It is noteworthy that the leukocyte levels in COVID-19 were significantly lower than those in the Negative and Other groups, which was consistent with Xiaoqing Liu et al.’s (44) claim that a viral infection could cause a decrease in the number of leukocytes, whereas bacteria typically produce an increase in leukocyte numbers.

The interpretability of deep learning models has been one of the main reasons why the performance of the models has been questioned. In the present study, our model was transparent, and the individual metrics were clinically meaningful and valuable, with leukocyte levels, age, neutrophil levels, total platelet volume, mean hemoglobin concentration and lymphocyte levels being the most important. In this study, we found that a decrease in platelet levels and a decrease in mean platelet volume were strongly correlated with the severity of the patients. In the study of Nishikawa M et al. showed a strong association between the concentration of platelet aggregates and the severity, mortality, respiratory status and level of vascular endothelial dysfunction in patients with COVID-19 (45). Thrombocytopenia has been reported in patients hospitalized with COVID-19, and lower platelet counts are associated with poorer clinical outcomes. A meta-analysis of 7,613 participants from 31 studies noted that lower platelet counts in severe COVID-19 infections were associated with a 3-fold increased risk of developing severe COVID-19 (46). In addition, individuals such as the elderly, men, those with a history of underlying disease, and those with blood type A have been reported to be at greater risk for SARS-CoV-2 or severe COVID-19 disease (47, 48). In addition, severe COVID-19 is characterized by lymphopenia, which may be caused by direct infection of lymphocytes or suppression of bone marrow by antiviral responses (49, 50).

Overall, our study presents 13 clinical indicators that are closely related to the course of disease progression in patients with COVID-19. In actual clinical monitoring, it is recommended that physicians in the emergency or intensive care unit pay closer attention to these indicators to be able to detect adverse conditions in patients in a timely manner. Nevertheless, there are still some problems in this study. For example, we lacked serial blood macroscopic measurements from the same COVID-19 patients to further confirm the causal relationship between platelet volume and disease severity. As well as, in this study, the cases of severe patients was limited, and the absence of predictors of mortality.

## Data availability statement

The original contributions presented in the study are included in the article/**Supplementary Material**. Further inquiries can be directed to the corresponding authors.

## Author contributions

XZ and YS participated in the development of the project and manuscript preparation. YS, JD, ZC and LL and MY performed data collection and analyses and interpreted the results. YS, JD, XL and XZ wrote the manuscript. XZ conducted this study. All authors contributed to the article and approved the submitted version.

## Supplementary material

Supplementary Figure 1 Statistical table of sample information. **(A)**, Distribution of the number of patients in different age groups and the number of patients with COVID-19 positive, COVID-19 negative, or other pneumonia. **(B)**, Distribution of total platelet volume and mean platelet volume in patients of different ages. **(C)**, Statistical bar chart of pathogens in patients with non-COVID-19 infections.
