# Supplementary figures and images for: Active regression model for clinical grading of COVID-19
Source: Front Immunol. 2023 Mar 21;14:1141996. doi: 10.3389/fimmu.2023.1141996 (PMC10071017; doi:10.3389/fimmu.2023.1141996)

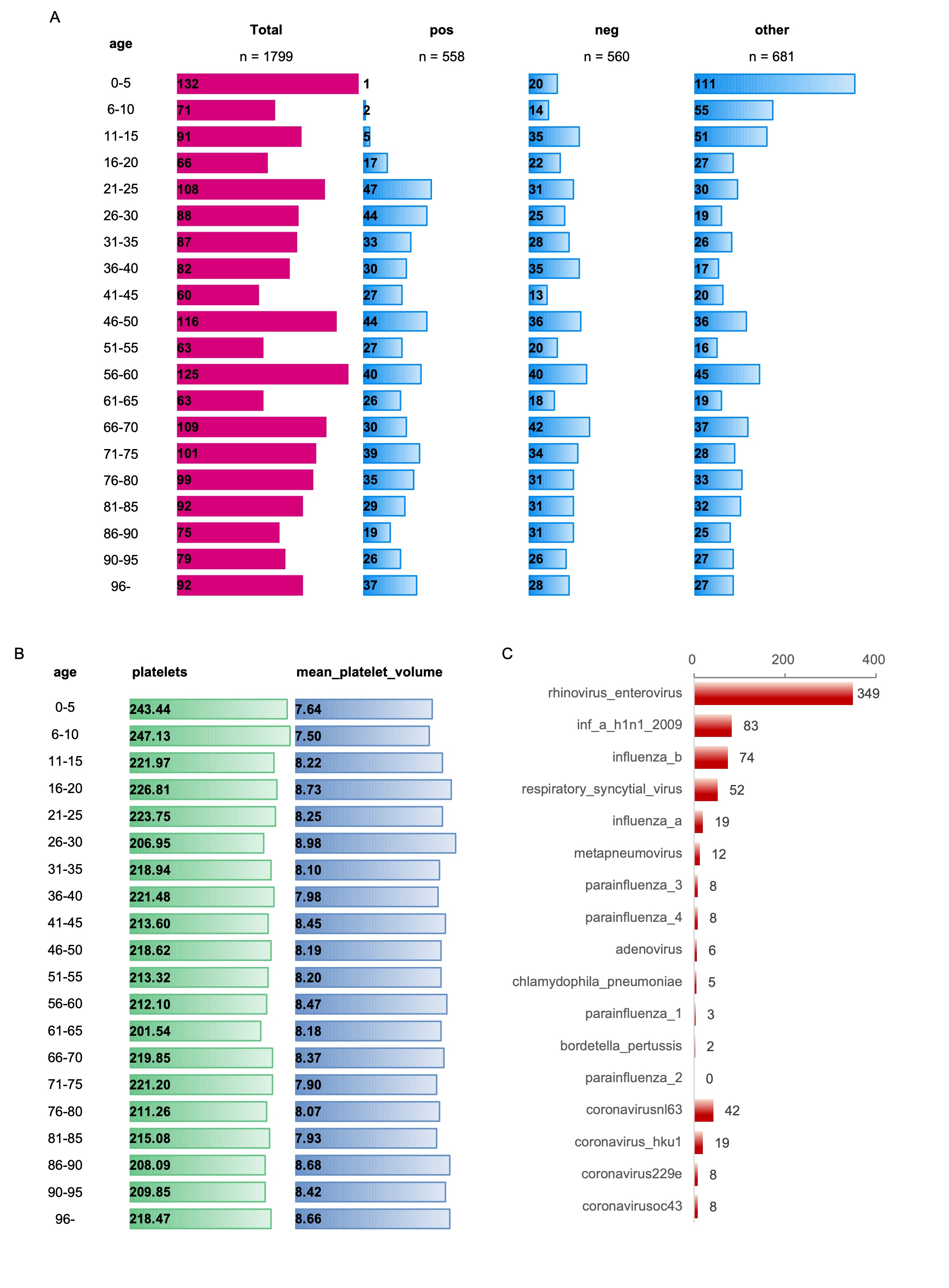

Supplement: Supplementary Figure 1 — Statistical table of sample information. (A), Distribution of the number of patients in different age groups and the number of patients with COVID-19 positive, COVID-19 negative, or other pneumonia. (B), Distribution of total platelet volume and mean platelet volume in patients of different ages. (C), Statistical bar chart of pathogens in patients with non-COVID-19 infections. [file Image_1.jpeg]
